# Clinical correlates of low-risk variants in *FGFR2*, *TNRC9*, *MAP3K1*, *LSP1 *and 8q24 in a Dutch cohort of incident breast cancer cases

**DOI:** 10.1186/bcr1793

**Published:** 2007-11-12

**Authors:** Petra EA Huijts, Maaike PG Vreeswijk, Karin HG Kroeze-Jansema, Catharina E Jacobi, Caroline Seynaeve, Elly MM Krol-Warmerdam, Pauline M Wijers-Koster, Jannet C Blom, Karen A Pooley, Jan GM Klijn, Rob AEM Tollenaar, Peter Devilee, Christi J van Asperen

**Affiliations:** 1 Department of Clinical Genetics, K5-R, Leiden University Medical Center, P.O. Box 9600, 2300 RC Leiden, The Netherlands; 2 Department of Human Genetics, S4-P, Leiden University Medical Center, P.O. Box 9600, 2300 RC Leiden, The Netherlands; 3 Department of Medical Decision Making, J10-S, Leiden University Medical Center, P.O. Box 9600, 2300 RC Leiden, The Netherlands; 4 Department of Medical Oncology, Family Cancer Clinic, Erasmus MC–Daniel den Hoed Cancer Center, P.O. Box 5201, 3008 AE Rotterdam, The Netherlands; 5 Department of Surgery, K6-R, Leiden University Medical Center, P.O. Box 9600, 2300 RC Leiden, The Netherlands; 6 Department of Pathology, L1-Q, Leiden University Medical Center, P.O. Box 9600, 2300 RC Leiden, The Netherlands; 7 Cancer Research UK, Department of Oncology, University of Cambridge, Strangeways Research Laboratory, Worts Causeway, Cambridge, CB1 8RN UK

## Abstract

**Introduction:**

Seven SNPs in five genomic loci were recently found to confer a mildly increased risk of breast cancer.

**Methods:**

We have investigated the correlations between disease characteristics and the patient genotypes of these SNPs in an unselected prospective cohort of 1,267 consecutive patients with primary breast cancer.

**Results:**

Heterozygote carriers and minor allele homozygote carriers for SNP rs889312 in the *MAP3K1 *gene were less likely to be lymph node positive at breast cancer diagnosis (*P *= 0.044) relative to major allele homozygote carriers. Heterozygote carriers and minor allele homozygote carriers for SNP rs3803662 near the *TNCR9 *gene were more likely to be diagnosed before the age of 60 years (*P *= 0.025) relative to major allele homozygote carriers. We also noted a correlation between the number of minor alleles of rs2981582 in *FGFR2 *and the average number of first-degree and second-degree relatives with breast cancer and/or ovarian cancer (*P *= 0.05). All other disease characteristics, including tumour size and grade, and oestrogen or progesterone receptor status, were not significantly associated with any of these variants.

**Conclusion:**

Some recently discovered genomic variants associated with a mildly increased risk of breast cancer are also associated with breast cancer characteristics or family history of breast cancer and ovarian cancer. These findings provide interesting new clues for further research on these low-risk susceptibility alleles.

## Introduction

Breast cancer is the most commonly occurring cancer among women in the industrialized world, where it accounts for 22% of all female cancers. Many breast cancer risk factors have been identified but a positive family history remains among the most important, with first-degree relatives of patients having an approximately twofold elevated risk [1]. Several genes are known to confer increased susceptibility to breast cancer [2], including *BRCA1 *and *BRCA2*, but it has been estimated that they explain only about 25% of the familial risk [3,4] and less than 5% of the total breast cancer incidence. This is because even though the risks conferred by mutations in *BRCA1 *and *BRCA2 *are substantial, the frequencies of these mutations in the population are very rare.

In contrast, twin studies and studies of the incidence of contralateral breast cancer have suggested that the proportion of breast cancer that can be attributed to genetic factors may be as high as 30% [5,6], implying that there are still breast cancer susceptibility genes to be identified. Linkage studies in multiple case families, however, have failed to identify further major breast cancer genes [7].

This failure has strengthened the idea that a large proportion of the familial risk of breast cancer is due to multiple loci, each conferring a small risk [4]. Variants in *CHEK2 *[8], *BRIP1 *[9] and *PALB2 *[10] fall into this category, but their population frequencies are very low, and thus so is their overall contribution to breast cancer incidence.

A recent large-scale, genome-wide scan for associations [11] has identified five new loci, each containing one or two SNPs that are very strongly associated with breast cancer in several populations of diverse ethnicity. Four of these contain plausible causative genes (*FGFR2*, *TNRC9*, *MAP3K1 *and *LSP1*). The per-allele odds ratios conferred by these variants ranged from 1.07 to 1.26, with minor allele frequencies ranging from 28% to 46%. These risks are too low to be useful in genetic predictive testing, but substantial proportions of breast cancer patients are heterozygous or are homozygous for these variants.

It is therefore interesting to investigate whether the identified variants in these loci are associated with particular disease characteristics, such as tumour grade and stage. Breast tumours arising in carriers of *BRCA1 *mutations have been shown to have a distinct phenotype [12,13], and patients carrying the 1100delC mutation in *CHEK2 *are at higher risk of developing secondary disease [14,15]. In a prospective hospital-based series of 1,267 incident breast cancer cases in the southwest of The Netherlands, we compared, in a case-only design, the genotypes at seven SNPs in the five newly identified low-risk loci with tumour and disease characteristics and other breast cancer risk factors.

## Materials and methods

### Patients

During the period October 1996–December 2005, patients who were diagnosed with a primary breast tumour were consecutively recruited from two academic hospitals (Leiden University Medical Center, Leiden and the Erasmus MC–Daniel den Hoed Cancer Center, Rotterdam) in the southwest of The Netherlands, irrespective of their family history. Patients over the age of 70 years at the time of diagnosis were excluded from the Erasmus MC–Daniel den Hoed Cancer Center cohort to enrich their population for hereditary factors. One hundred and three patients who were invited to join this study declined to participate. The final Dutch hospital-based ORIGO cohort of breast cancer patients consisted of 1,359 patients, of which 1,207 were diagnosed with invasive breast cancer and 152 with carcinoma *in situ*.

After giving informed consent, patients filled in a questionnaire on breast cancer risk factors (age at menarche and age at first pregnancy, total number of pregnancies and duration of breastfeeding, age at menopause, use of oral contraceptives, alcohol use) and on family cancer history. Questionnaires were not returned by 90 of the 1,359 patients (6.6%). A further two patients were excluded from the analysis because of poor genotyping quality, so that for a total number of 1,267 patients in the ORIGO cohort both genotyping information and information on personal breast cancer risk factors were known. This final cohort consisted of 549 patients from the Leiden University Medical Center and 718 patients from the Erasmus MC–Daniel den Hoed Cancer Center.

Age at diagnosis and body mass index were recorded from the hospital records, and information on tumour histology, including tumour size and lymph node status, and on oestrogen receptor, progesterone receptor and HER2/*neu *status, was retrieved from the pathology reports. Because of the small number of patients for whom HER2/*neu *status was known (*n *= 76), this characteristic was excluded from our statistical analysis. A blood sample was collected from each patient and DNA was isolated. During the ongoing follow-up, local, locoregional and distant recurrence of disease and death were recorded.

To assess allele frequencies in the general population, we genotyped 378 healthy blood donors from the southwest region of The Netherlands (93 females, 285 males), and 234 healthy spouses of family members of families with multiple breast cancer cases (169 males and 65 females) [16].

The Medical Ethical Review Boards of the involved centres approved the study protocol, under the condition that written informed consent was obtained from the patients.

### Genotyping

With the ORIGO cohort we participated in the third stage of the Breast Cancer Association Consortium (BCAC) genome-wide association study, in which 30 of the most significant SNPs from stage 2 were genotyped in 22 case–control studies [11]. In total these studies comprised 21,668 cases of invasive breast cancer, 967 cases of carcinoma *in situ *and 20,973 control cases [11]. A 31st SNP was added later as a result of fine-scale mapping of the *TNRC9 *locus. Details on the genotyping methods can be found in the article by Easton and colleagues [11]. In the ORIGO cohort, genotypes were successfully called in at least 95% of the samples for all SNPs – except for SNP rs8051542, for which successful genotype calling was present in 89.5% of all samples.

### Statistical analysis

The prevalence of the minor allele of each SNP in the ORIGO population was compared with the prevalence in the control population using chi-square tests. The odds ratios were calculated using regression analysis.

The combined effect of SNP rs2981582 and each one of the other six SNPs was explored pairwise in the ORIGO cohort. Patients were stratified into nine groups according to their genotype at rs2981582 and at the other SNP, and the odds ratios of each subgroup were calculated using regression analysis – the group of patients being homozygous for the major alleles of both SNPs serving as reference category (odds ratio = 1.0). Analysis of three or more SNPs at the same time resulted in subgroups too small for statistical analysis.

In the BCAC study, the odds ratios for heterozygous and homozygous carriers of the rare allele of each SNP were in agreement with a codominant model. We therefore analysed the effect of each SNP on disease characteristics by classifying the patients from our ORIGO cohort into three groups: patients homozygous for the major allele (wt/wt), patients heterozygous for the wildtype and mutated or minor allele (wt/mt), and patients homozygous for the minor allele (mt/mt). Since the group of patients homozygous for the minor allele was often small, associations between the SNP status and the disease characteristics were also studied comparing the group of patients homozygous for the major allele with those carrying one or two minor alleles.

Disease characteristics were compared between the three patient groups using logistic regression for dichotomous variables, chi-square tests for categorical variables, and one-way analyses of variance for continuous variables. For the analyses comparing two patient groups, Pearson's correction was applied to the chi-square tests and continuous variables were compared using the Student *t *test.

*P *< 0.05 was considered statistically significant. *P *< 0.1 was considered a possible trend that could be explored further in larger study groups.

All analyses on the ORIGO cohort and its control group were performed with SPSS version 11.0.1 (SPSS Inc., Chicago, IL, USA).

## Results

The characteristics of the 1,267 patients in the ORIGO cohort are summarized in Table 1. The average age at diagnosis was 54.0 years, which is several years younger than the average for all Dutch patients (61.3 years) [17]. This younger diagnosis age is due to the fact that in one accrual hospital only patients diagnosed before the age of 70 years were included. All other disease characteristics are not significantly different from population-based data.

**Table 1:** Baseline characteristics of the Dutch hospital-based ORIGO cohort of breast cancer patients (*n *= 1267)

Variable	Value
Age at diagnosis (years) (*n *= 1,254)	54 ± 11.2 (21–88)
Follow-up time (months) (*n *= 476)	64 ± 31 (3–126)
Body mass index (kg/m^2^) (*n *= 1,203)	25 ± 4 (16–45)
Sex (*n *= 1,267)	
Female	1262 (99.6%)
Male	5 (0.4%)
Bilaterality of breast cancer^a ^(*n *= 1,265)	
Bilateral	79 (6.2%)
Unilateral	1186 (93.8%)
Tumour characteristics	
Bloom–Richardson grade (*n *= 964)	
Grade I	175 (18.2%)
Grade II	399 (41.4%)
Grade III	390 (40.5%)
Clinical staging of cancer (UICC)^b ^(*n *= 1,230)	
Stage 0 (*in situ*)	144 (11.7%)
Stage 1	479 (38.9%)
Stage 2	526 (42.8%)
Stage 3	81 (6.6%)
Receptor status	
Oestrogen receptor (*n *= 831)	
Positive	597 (71.8%)
Negative	234 (28.2%)
Progesterone receptor (*n *= 683)	
Positive	399 (58.4%)
Negative	284 (41.6%)
Human epidermal growth factor receptor 2 (HER2/*neu*) (*n *= 76)	
Positive	14 (18.4%)
Negative	62 (81.6%)
Risk factors	
Family history^c ^(*n *= 1,253)	
Negative	753 (60.1%)
Positive	500 (39.9%)
Pregnancy (*n *= 1,179)	
Ever	968 (82.1%)
Never	211 (17.9%)
Breastfeeding (*n *= 1,215)	
Ever	693 (57%)
Never	522 (43%)
Breastfeeding duration (months)	5.21 ± 9.8 (0–121)

Data presented as the mean ± standard deviation (range) or number (% of total number). ^a^Within 6 months of first diagnosis. ^b^International Union Against Cancer (UICC) stages, based on T classification and N classification, known in 1,230 patients. ^c^Family history is considered positive when at least one first-degree or second-degree relative also had breast cancer, irrespective of age at onset.

The ORIGO cohort took part in a large three-phase, genome-wide study conducted by the BCAC, which identified seven SNPs to be significantly associated with breast cancer [11]. These SNPs map to five genomic loci, including the *FGFR2 *gene (SNP rs2981582) that encodes the fibroblast growth factor receptor 2, the *TNRC9 *gene (rs12443621, rs8051542, rs3803662), the *MAP3K1 *gene (rs889312) that encodes mitogen-activated protein kinase kinase kinase 1, the *LSP1 *gene (rs3817198) that codes for lymphocyte-specific protein 1, and a gene-less linkage disequilibrium block of ~110 kb on 8q24 (rs13281615).

Table 2 presents these seven SNPs with the strengths of the associations in the total BCAC study, as compared with those in the ORIGO cohort alone. The ORIGO cohort and the control cohort were in Hardy–Weinberg equilibrium for each of the seven SNPs investigated. Only the SNP in *FGFR2 *reached formal statistical significance in the ORIGO cohort (*P *trend < 0.007), in the same direction as in the overall BCAC study. The other SNPs did not reach statistical significance, although the observed per-allele odds ratios were similar to those obtained in the overall BCAC study, with the exception of SNP rs889312.

**Table 2:** Minor allele frequencies, *P *values and per-allele odds ratios of the seven SNPs in the Breast Cancer Association Consortium (BCAC) study and in the Dutch hospital-based ORIGO cohort of breast cancer patients

SNP	Minor allele frequency		*P *trend		Per-allele odds ratio	
	
	BCAC study	ORIGO cohort	BCAC study	ORIGO cohort	BCAC study	ORIGO cohort
rs2981582	0.38	0.38	2.10^-76^	0.007	1.26	1.30
rs3803662	0.25	0.26	1.10^-36^	0.131	1.20	1.13
rs889312	0.28	0.31	7.10^-20^	0.727	1.13	1.03
rs12443621^a^	0.46	0.43	2.10^-19^	0.805	1.11	1.05
rs8051542^a^	0.44	0.48	1.10^-12^	0.494	1.09	0.96
rs13281615	0.40	0.39	5.10^-12^	0.277	1.08	1.10
rs3817198	0.30	0.30	3.10^-9^	0.529	1.07	1.06

^a^SNPs rs12443621 and rs8051542 were in linkage disequilibrium with SNP rs3803662.

An important issue to be resolved is whether women carrying risk alleles at more than one of these five loci are at an even greater risk of developing breast cancer than those carrying only one risk allele, and whether there are epistatic interactions. In an attempt to address this issue, we analysed the combined effects of SNP rs2981582 in *FGFR2 *and each one of the other six SNPS on breast cancer risk in our ORIGO cohort (Additional file 1). A significant stepwise increase of the odds ratio was observed in the ORIGO cohort, depending on the combined number of minor alleles present for rs2981582 (*FGFR2*) and for rs3803662 (*TNRC9*) (*P *= 0.022; Table 3), with an observed maximum odds ratio of 2.22.

**Table 3:** Combined odds ratios for the two most significant SNPs in the Dutch hospital-based ORIGO cohort of breast cancer patients

SNP rs2981582 in *FGFR2*	SNP rs3803662 near *TNRC9*		
	
	0 (wt/wt)	1 (wt/mt)	2 (mt/mt)
0 (wt/wt)	1.00	1.25 (0.87–1.78)	1.12 (0.64–1.97)
1 (wt/mt)	1.28 (0.95–1.72)	1.48 (1.06–2.05)	1.90 (1.07–3.39)
2 (mt/mt)	1.76 (1.17–2.66)	2.22 (1.37–3.60)	1.35 (0.57–3.20)

The combined effect of SNPs rs2981582 and rs3803662 on breast cancer risk was studied in the ORIGO cohort. The observed odds ratios and their 95% confidence intervals are presented. wt/wt, patients homozygous for the wildtype allele; wt/mt, patients heterozygous for the wildtype and mutated or minor allele; mt/mt, patients homozygous for the mutated or minor allele. *P *value for overall differences = 0.022.

The main objective of our current study was to investigate associations between disease characteristics and the SNP genotypes in the ORIGO cohort. Significant (*P *< 0.05) and near-significant results (*P *< 0.10) are presented in Table 4 (see also Additional file 2).

**Table 4:** Associations of SNPs with disease characteristics in the Dutch hospital-based ORIGO cohort of breast cancer patients

Patient or disease characteristics	wt/wt	wt/mt	mt/mt	*P *value, three groups^a^	*P *value, two groups^b^
SNP rs2981582 (in *FGFR2*)					
Family history					
Positive for breast cancer (%)	36	40	45	0.089	0.095
Positive for breast cancer and/or ovarian cancer (%)	38	42	48	0.065	0.089
Average number of relatives with breast cancer or ovarian cancer divided by the total number of female relatives	0.067	0.071	0.089	0.05	0.193
Average duration of breastfeeding (months)	4.5	5.5	5.8	0.224	0.062
SNP rs3817198 (in *LSP1*)					
Positive lymph-node status (%)	39	44	44	0.190	0.078
SNP rs889312 (near *MAP3K1*)					
Positive lymph-node status (%)	45	40	36	0.090	0.044
SNP rs3803662 (near *TNRC9*)					
Age at diagnosis <60 years (%)^c^	68	73	82	0.054	0.025
Family history					
Average number of first-degree relatives with breast cancer	0.23	0.28	0.31	0.156	0.072
Average number of relatives with breast cancer divided by total number of female relatives	0.063	0.070	0.082	0.166	0.118
Average duration of breastfeeding (months)	4.9	5.7	6.4	0.206	0.097

wt/wt, patients homozygous for the wildtype allele; wt/mt, patients heterozygous for the wildtype and mutated or minor allele; mt/mt, patients homozygous for the mutated or minor allele. ^a^Comparing the three genotype groups separately. ^b^A codominant model, comparing the group of homozygous patients for the wildtype allele with the combined group of heterozygous patients and patients homozygous for the minor allele. ^c^See also Figure 1.

In the ORIGO cohort, the minor allele of SNP rs2981582 in *FGFR2 *was found to correlate with a positive family history of breast cancer, defined as the ratio of the number of relatives with breast cancer and/or ovarian cancer relative to the total number of female relatives. This ratio, which corrects for differences in family size, was higher for carriers of one or two of the minor alleles (*P *= 0.05). Nonsignificant trends for association were observed for other definitions, such as the presence of at least one first-degree or second-degree family member with breast cancer, or with breast cancer and/or ovarian cancer. Likewise, the minor allele of SNP rs3803662 in the *TNRC9 *locus also showed a nonsignificant trend with a larger number of first-degree relatives with breast cancer (on average, 0.23 in major allele homozygous patients versus 0.28 and 0.31 for heterozygous patients and minor allele homozygous patients, respectively).

In our cohort, the minor allele of SNP rs3803662 in the *TNRC9 *locus was associated with younger age at diagnosis, reflected by an overrepresentation of carriers of one or two minor alleles in the age groups between 40 years and 60 years (Figure 1). The mean age at diagnosis was 54.3 years, 53.4 years and 52.5 years for the patients homozygous for the major allele, for heterozygous patients and for patients homozygous for the minor allele, respectively, but the difference between these values was not significant (*P *= 0.199).

**Figure 1:**
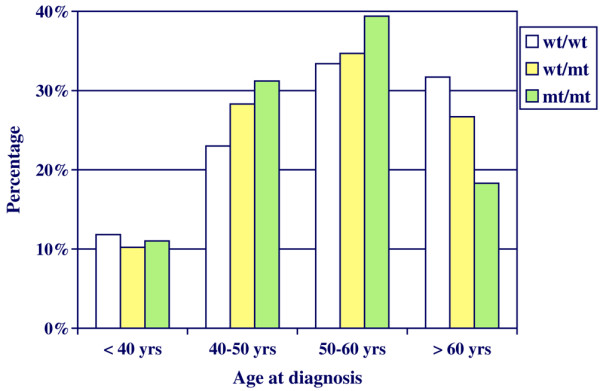
Age at breast cancer diagnosis according to rs3803663 (*TNRC9*) genotype in the ORIGO cohort. The age at diagnosis in the Dutch hospital-based ORIGO cohort of breast cancer patients is represented by four age categories: before the age of 40 years, between the ages of 40 years and 50 years, between the ages of 50 years and 60 years, and above the age of 60 years. wt/wt, patients homozygous for the wildtype or major allele; wt/mt, patients heterozygous for the wildtype and mutated or minor allele; mt/mt, patients homozygous for the mutant or minor allele.

Heterozygote carriers and minor allele homozygote carriers for SNP rs889312 in the *MAP3K1 *gene were less likely to have lymph-node-positive breast cancer at diagnosis (*P *= 0.044) compared with major allele homozygote carriers. An opposite but nonsignificant trend was observed for SNP rs3817198; a slightly higher proportion of heterozygous patients and minor allele homozygous patients had lymph-node-positive breast cancer compared with major allele homozygous patients (*P *= 0.078).

In the ORIGO cohort, information on pregnancy and breastfeeding was present for 1,179 patients. Of these, 690 patients had provided breastfeeding for more than 1 month in total. Although not reaching significance, homozygous carriers and heterozygous carriers of the minor allele of SNP rs2981582 had breastfed for a longer period of time than homozygous carriers of the major allele (5.8 months and 5.5 months versus 4.5 months, respectively). The same trend was seen in homozygous carriers and heterozygous carriers of the minor allele of rs3803662, who had breastfed for a longer period of time than homozygous carriers of the major allele (6.4 months, 5.7 months and 4.9 months, respectively).

The tumours of heterozygous carriers or homozygous carriers of the minor allele of SNP rs12443621, which is in strong linkage disequilibrium with SNPs rs8051542 and rs3803662 in the *TNRC9 *locus, were significantly more often negative for the progesterone receptor (41.4% and 52.3% for the heterozygous patients and for homozygous patients of the minor allele versus 38.2% in the homozygous patients for the major allele, *P *= 0.048). The same trend was seen with SNPs rs8051542 and rs3803662, but did not reach significance (*P *= 0.3 and *P *= 0.69, respectively). No such associations were observed for the oestrogen receptor status.

The minor allele of SNP rs12443621 near *TNRC9 *was also significantly associated with a slightly lower body mass index (*P *= 0.043). A similar lower body mass index was also seen in carriers of the minor allele of nearby SNP rs3803662, but the association was not significant (*P *= 0.680). SNP rs8051542, in the same region, did not show the same trend and did not reach significance.

Follow-up was updated in October 2006 for 476 patients diagnosed in the Leiden University Medical Center Hospital, with a mean follow-up period of 64 months, but no significant associations were observed between overall survival and disease-free survival and any of the seven SNPs.

No differences were observed between the genotype groups for any of the seven SNPs tested and the bilateral occurrence of breast cancer, the Bloom–Richardson differentiation grade, carcinoma *in situ *versus invasive cancer, the clinical breast cancer stage (International Union Against Cancer), age at first pregnancy or the total number of pregnancies and total duration of pregnancies.

## Discussion

Even though our ORIGO cohort is a hospital-based cohort, the patient and tumour characteristics of the included patients are very similar to those of a population-based breast cancer cohort, with the exception of age at diagnosis [18-20]. The percentage of patients with a positive family history for breast cancer is not different from other breast cancer populations [1,21].

In the ORIGO cohort, we first studied the association between seven recently identified low-risk breast cancer susceptibility alleles in five genomic regions and breast cancer risk in general.

It is interesting to note that, although the ORIGO cohort consists of only 1,359 cases, the per-allele odds ratios calculated for the seven SNPs in the BCAC study and in the ORIGO cohort are similar. Although the increase in breast cancer risk inferred by the minor allele of each of these SNPs is only small, the attributable risks are high due to the high frequency of the minor allele in the population.

To investigate the combined effects of these SNPs on breast cancer risk, we calculated the odds ratios of the combination of the most important SNP (rs2981582 in *FGFR2*) with one other SNP. We found significantly higher odds ratios when genotypes at SNP rs2981582 (*FGFR2*) and at SNP rs3803662 (*TNRC9*) were combined, with magnitudes in keeping with a simple multiplicative model. If confirmed in larger cohorts, this statistical interaction could provide clues about the biological functions of the involved proteins in breast cancer development.

In the present study, we proceeded by focusing on the associations between patient characteristics and histopathological characteristics and genotypes at the seven SNPs in our ORIGO cohort. Although this cohort is relatively small, it is also homogeneous and extensively characterized, allowing an elaborate first exploration of the possible associations between the SNPs and disease characteristics.

In the BCAC study, SNP rs2981582 in *FGFR2 *was most strongly associated with breast cancer [11], an association that was replicated in three independent postmenopausal breast cancer cohorts [22]. In the ORIGO cohort, rs2981582 genotypes correlated with a positive family history of breast cancer and/or ovarian cancer, although this was significant only when the ratio of the number of relatives with breast cancer and/or ovarian cancer relative to the total number of female relatives was compared. The BCAC study also found an association between this SNP and women with a first-degree relative with breast cancer. Our results thus suggest that this association may also include relatives with ovarian cancer. Expression of *FGFR2 *subtype IIIb has been implicated to be upregulated in up to 80% of epithelial ovarian cancers [23,24]. Although the functionality of rs2981582 with regard to an increased cancer risk is as yet unresolved, it has been speculated that it is mediated through regulation of *FGFR2 *expression [11].

Two other SNPs (rs3803662 and rs13281615) were also linked to family history in the BCAC study, and this was also noted for rs3803662 in the ORIGO cohort, although not significantly (*P *= 0.072). This effect was observed only for breast cancer family history, and was lost when adding ovarian cancer cases to the analysis. The association between rs13281615 and family history was not observed in the ORIGO cohort.

We did not confirm the association between rs3803662 in *TNRC9*, the second most significant SNP in the BCAC study, and breast cancer risk in our cohort. However, we did observe that heterozygote carriers and minor allele homozygote carriers for this SNP had a higher probability of being diagnosed with a breast tumour before age 60 years than major allele homozygote carriers. This effect is to be expected for susceptibility loci, although it was not seen for other SNPs in our cohort, and only a trend was observed in the BCAC study [11].

It is possible that a low-risk allele not only influences the chance of developing breast cancer but also influences tumour characteristics such as invasiveness. In our cohort we found that patients carrying one or two minor alleles of rs889312 in *MAP3K1 *were less likely to have lymph-node-positive breast cancer, suggesting that the invasive potential of the tumour in these patients might be lower. A trend for an opposite effect was observed for rs3817198 in *LSP1*. There was, however, no significant effect of either one of these SNPs on overall survival or disease-free survival, but this may be due to the relatively small sample size and the short period of follow-up.

A prolonged period of breastfeeding is a known protective factor against breast cancer [25]. Carriers of one or two minor alleles of SNPs rs2981582 and rs3803662 breastfed for a longer period of time, but developed breast cancer nonetheless, suggesting that the protective effect of breastfeeding might have been counteracted by the presence of the minor allele of either SNP.

We have shown previously that the CHEK2*1100delC germline mutation was more prevalent among patients with a positive oestrogen receptor status in the ORIGO cohort [26]. A similar finding was made for SNP rs3803662, in that the risk of this allele was strongly confined to patients with oestrogen-receptor-positive tumours [27]. We examined this association for the seven SNPs in our cohort but did not find any effect with oestrogen receptor status for any, and only a few weak associations were found with progesterone receptor status. It is conceivable that the risks conferred by these alleles are expressed only in receptor-positive or receptor-negative subgroups of patients, but these differences in risk are probably too small to be detected in our cohort. With the current cohort of 831 patients with known oestrogen receptor status, we had only a statistical power of 59% to detect a difference in oestrogen receptor status of 10%.

We did not observe any association between genotypes at the seven SNPs and a variety of other clinical parameters, including the bilateral occurrence of breast cancer, the Bloom–Richardson grade, the clinical International Union Against Cancer stage, age at first pregnancy or the total number of pregnancies and duration of pregnancies.

## Conclusion

In summary, several of the seven recently identified common genetic variants that predispose to breast cancer are associated with certain clinical characteristics, such as family history, nodal involvement, and age at diagnosis, in a Dutch cohort of breast cancer patients. These findings should be regarded with caution, given the relatively small size of the ORIGO cohort. The clinical implications of these findings are at present therefore undetermined. These results do, however, provide interesting new clues for further association studies in larger patient cohorts as well as for further research aimed at elucidating the causal relationship of the SNPs with breast cancer.

## Abbreviations

BCAC = Breast Cancer Association Consortium; ORIGO = Dutch hospital-based cohort of breast cancer patients; SNP = single nucleotide polymorphism.

## Competing interests

The authors declare that they have no competing interests.

## Authors' contributions

PEAH carried out data curation and statistical analyses, and drafted the manuscript. MPGV, KHGK-J and PMW-K isolated DNA and prepared microtitre array plates for genotyping. CEJ maintained the patient database and supervised statistical analyses. CS, EMMK-W and JCB carried out patient accrual and collected questionnaire data. KAP performed the genotyping. JGMK, RAEMT, PD and CJvA conceived of the study, obtained financial support, participated in the design and coordination of the study, and helped to draft the manuscript. All authors read and approved the final manuscript.

## Supplementary Material

Additional file 1 A pdf file containing a table that presents the combined odds ratios for rs2981582 and for each of the other investigated SNPs in the ORIGO cohort. The combined effect on the breast cancer risk of SNP rs2981582 and each of the other six SNPs was explored pairwise. The observed odds ratios and their 95% confidence intervals are presented in this additional file. Click here for file

Additional file 2 A pdf file containing a table that presents the associations of each of the seven investigated SNPs with disease characteristics and patient characteristics in the ORIGO cohort. Click here for file
