# Supplementary material for: Clinical correlates of low-risk variants in FGFR2, TNRC9, MAP3K1, LSP1 and 8q24 in a Dutch cohort of incident breast cancer cases
Source: Breast Cancer Res. 2007 Nov 12;9(6):R78. doi: 10.1186/bcr1793 (PMC2246176; doi:10.1186/bcr1793)
Supplement: Additional file 1 — A pdf file containing a table that presents the combined odds ratios for rs2981582 and for each of the other investigated SNPs in the ORIGO cohort. The combined effect on the breast cancer risk of SNP rs2981582 and each of the other six SNPs was explored pairwise. The observed odds ratios and their 95% confidence intervals are presented in this additional file. [file bcr1793-S1.pdf]

Supplemental table 1 Combined ORs for rs2981582 and each of the other investigated SNPs in the ORIGO cohort

		**rs3803662 near TNRC9**		
		0 (wt/wt)	1 (wt/mt)	2 (mt/mt)
**rs2981582 in FGFR2**	0 (wt/wt)	1.00	1.25 (0.87-1.78)	1.12 (0.64-1.97)
	1 (wt/mt)	1.28 (0.95-1.72)	1.48 (1.06-2.05)	1.90 (1.07-3.39)
	2 (mt/mt)	1.76 (1.17-2.66)	2.22 (1.37-3.60)	1.35 (0.57-3.20)

P-value for overall differences was p=0.022

		**rs12443621 near TNRC9**		
		0 (wt/wt)	1 (wt/mt)	2 (mt/mt)
**rs2981582 in FGFR2**	0 (wt/wt)	1.00	1.14 (0.78-1.66)	1.14 (0.70-1.86)
	1 (wt/mt)	1.26 (0.85-1.85)	1.44 (1.00-2.07)	1.65 (1.04-2.64)
	2 (mt/mt)	2.12 (1.22-3.69)	2.04 (1.27-3.27)	1.33 (0.74-2.40)

P-value for overall differences was p=0.018

		**rs8051542 near TNRC9**		
		0 (wt/wt)	1 (wt/mt)	1 (mt/mt)
**rs2981582 in FGFR2**	0 (wt/wt)	1.00	1.14 (0.76-1.71)	0.98 (0.61-1.57)
	1 (wt/mt)	1.34 (0.87-2.05)	1.40 (0.95-2.07)	1.20 (0.77-1.87)
	2 (mt/mt)	1.93 (1.07-3.47)	1.78 (1.09-2.91)	1.50 (0.80-2.79)

P-value for overall differences was p=0.124

		**rs889312 near MAP3K1**		
		0 (wt/wt)	1 (wt/mt)	2 (mt/mt)
**rs2981582 in FGFR2**	0 (wt/wt)	1.00	0.76 (0.54- 1.07)	0.73 (0.42-1.29)
	1 (wt/mt)	1.00 (0.73-1.37)	1.08 (0.79-1.47)	1.61 (0.93-2.80)
	2 (mt/mt)	1.25 (0.82-1.90)	1.64 (1.04-2.58)	1.63 (0.68-3.90)

P-value for overall differences was p=0.018

		rs13281615 on 8q24		
		0 (wt/wt)	1 (wt/mt)	2 (mt/mt)
**rs2981582 in FGFR2**	0 (wt/wt)	1.00	1.12 (0.78-1.61)	1.10 (0.67-1.80)
	1 (wt/mt)	1.28 (0.89-1.84)	1.48 (1.05-2.08)	1.33 (0.83-2.12)
	2 (mt/mt)	1.29 (0.79-2.11)	2.23 (1.39-3.57)	2.18 (1.17-4.05)

P-value for overall differences was p=0.018

		rs3817198 in LSP1		
		0 (wt/wt)	1 (wt/mt)	2 (mt/mt)
**rs2981582 in FGFR2**	0 (wt/wt)	1.00	1.16 (0.82-1.63)	1.15 (0.64-2.04)
	1 (wt/mt)	1.28 (0.94-1.74)	1.54 (1.12-2.12)	1.31 (0.79-2.17)
	2 (mt/mt)	1.85 (1.22-2.81)	1.92 (1.21-3.04)	1.37 (0.65-2.89)

P-value for overall differences was p=0.044

			rs889312 near MAP3K1		
		**rs3803662 near TNRC9**	0 (wt/wt)	1 (wt/mt)	2 (mt/mt)
**rs2981582 in FGFR2**	0 (wt/wt)	0 (wt/wt)	1.00	0.79 (0.49-1.30)	1.07 (0.48-2.38)
		1 (wt/mt)	1.26 (0.76-2.09)	1.11 (0.64-1.94)	0.66 (0.26-1.68)
		2 (mt/mt)	1.81 (0.77-4.27)	0.54 (0.21-1.37)	0.72 (0.15-3.32)
	1 (wt/mt)	0 (wt/wt)	1.11 (0.72-1.72)	1.07 (0.69-1.64)	1.93 (0.89-4.20)
		1 (wt/mt)	1.18 (0.72-1.93)	1.39 (0.85-2.27)	1.68 (0.71-3.98)
		2 (mt/mt)	1.12 (0.51-2.48)	2.22 (0.91-5.42)	5 cases
	2 (mt/mt)	0 (wt/wt)	1.22 (0.69-2.15)	2.00 (1.03-3.88)	2.32 (0.63-8.52)
		1 (wt/mt)	1.90 (0.90-4.02)	2.19 (1.07-4.48)	1.79 (0.47-6.78)
		2 (mt/mt)	2.14 (0.58-7.94)	0.54 (0.15-1.94)	One case

P-value for overall differences was p=0.209

The combined effect on breast cancer risk of SNP rs2981582 and each one of the other six SNPs was explored pairwise in the ORIGO cohort. The observed odds ratios and their 95% confidence intervals are given in these tables.
Abbreviations: SNP, single nucleotide polymorphism; wt/wt, homozygotes for the wildtype allele; wt/mt, heterozygotes; mt/mt, homozygotes for the mutated allele.
