# Supplementary material for: Clinical correlates of low-risk variants in FGFR2, TNRC9, MAP3K1, LSP1 and 8q24 in a Dutch cohort of incident breast cancer cases
Source: Breast Cancer Res. 2007 Nov 12;9(6):R78. doi: 10.1186/bcr1793 (PMC2246176; doi:10.1186/bcr1793)
Supplement: Additional file 2 — A pdf file containing a table that presents the associations of each of the seven investigated SNPs with disease characteristics and patient characteristics in the ORIGO cohort. [file bcr1793-S2.pdf]

Supplemental table 2 Associations of SNPs with disease characteristics in the ORIGO cohort

SNP: rs2981582 in FGFR2

Patient or disease characteristics		n=	Phenotype per genotype			p-value 3 groups[1]	p-value 2 groups[2]
			wt/wt	wt/mt	mt/mt		
Genotype (number of patients)			406	645	258		
Age at diagnosis (average)		1207	53.50	53.92	54.66	0.466	0.370
Age groups at diagnosis (%)	<40 years	1206	11.8%	10.2%	12.3%	0.758	0.616
	40-50 years		26.9%	25.4%	22.6%		
	50-60 years		34.7%	35.1%	33.6%		
	>60 years		26.6%	29.4%	31.5%		
BMI (average)		1164	25.26	25.33	25.34	0.972	0.810
Bilateral breast cancer present (%)		1218	5.9%	6.5%	7.1%	0.814	0.599
Stage of primary tumor (%)	T0 (in situ)	1193	10.5%	9.0%	8.9%	0.033	0.363
	T1		56.9%	52.6%	63.6%		
	T2		24.5%	30.5%	25.0%		
	T3 or T4		8.1%	7.9%	2.5%		
Lymph nodes involved (stage N1, N2 or N3) (%)		1080	41.9%	40.9%	41.0%	0.955	0.960
Clinical UICC stage (%)	1	1046	45.0%	42.4%	48.3%	0.082	0.592
	2		46.6%	49.1%	48.8%		
	3		8.4%	8.4%	2.9%		
Bloom-Richardson grade (%)	1	928	17.2%	19.1%	19.1%	0.514	0.340
	2		39.3%	41.3%	45.5%		
	3		43.5%	39.6%	35.4%		
Progesterone receptor positive (%)		668	61.3%	53.5%	63.6%	0.063	0.254
Estrogen receptor positive (%)		804	71.5%	71.1%	75.0%	0.622	0.831
Family history positive for breast cancer (%)		1205	36.3%	40.2%	45.3%	0.089	0.095
Family history positive for breast and/or ovarian cancer (%)		1205	38.2%	42.0%	47.9%	0.065	0.089
Number of relatives with breast cancer divided by total number of female relatives (average)		1205	0.061	0.066	0.082	0.062	0.164
Number of relatives with breast or ovarian cancer divided by total number of female relatives (average)		1205	0.067	0.071	0.089	0.050	0.193
Number of first degree relatives with breast cancer (average)		1205	0.24	0.23	0.32	0.094	0.611
Ever pregnant (%)		1134	83.5%	81.9%	82.3%	0.825	0.544
Age at first pregnancy (average)		927	24.72	25.12	25.42	0.238	0.130
Total duration of pregnancies (average, in months)		1168	14.68	15.44	14.81	0.662	0.496
Breast feeding (%)		1168	57.9%	56.2%	59.1%	0.713	0.775
Total duration of breast feeding (average, in months)		1168	4.54	5.51	5.83	0.224	0.062
Died during follow-up (%)		486	16.9%	17.3%	16.8%	0.991	0.942

SNP: rs3803662 near TNRC9

Patient or disease characteristics		n=	Phenotype per genotype			p-value 3 groups[1]	p-value 2 groups[2]
			wt/wt	wt/mt	mt/mt		
Genotype (number of patients)			672	495	120		
Age at diagnosis (average)		1184	54.30	53.39	52.54	0.199	0.097
Age groups at diagnosis (%)	<40 years	1184	11.8%	10.2%	11.0%	0.054	0.025
	40-50 years		23.0%	28.3%	31.2%		
	50-60 years		33.4%	34.7%	39.4%		
	>60 years		31.7%	26.7%	18.3%		
BMI (average)		1143	25.39	25.20	25.06	0.680	0.403
Bilateral breast cancer present (%)		1195	6.5%	6.4%	5.5%	0.927	0.848
Stage of primary tumor (%)	T0 (in situ)	1170	7.8%	10.7%	12.1%	0.525	0.289
	T1		57.3%	55.3%	47.7%		
	T2		27.3%	27.7%	32.7%		
	T3 or T4		7.6%	6.3%	7.4%		
Lymph nodes involved (stage N1, N2 or N3) (%)		1060	40.3%	43.9%	36.7%	0.335	0.468
Clinical UICC stage (%)	1	1027	45.6%	42.1%	45.2%	0.781	0.586
	2		46.7%	50.8%	46.2%		
	3		7.7%	7.2%	8.6%		
Bloom-Richardson grade (%)	1	910	17.1%	20.7%	19.5%	0.758	0.412
	2		42.3%	39.7%	41.5%		
	3		40.6%	39.7%	39.0%		
Progesterone receptor positive (%)		658	59.8%	57.4%	54.0%	0.678	0.445
Estrogen receptor positive (%)		786	72.3%	72.0%	72.1%	0.996	0.935
Family history positive for breast cancer (%)		1183	39.0%	40.3%	43.5%	0.656	0.493
Family history positive for breast and/or ovarian cancer (%)		1183	41.2%	42.1%	44.4%	0.813	0.642
Number of relatives with breast cancer divided by total number of female relatives (average)		1183	0.063	0.070	0.082	0.166	0.118
Number of relatives with breast or ovarian cancer divided by total number of female relatives (average)		1183	0.070	0.075	0.086	0.362	0.256
Number of first degree relatives with breast cancer (average)		1183	0.23	0.28	0.31	0.156	0.072
Ever pregnant (%)		1115	80.6%	84.1%	83.8%	0.323	0.133
Age at first pregnancy (average)		911	25.16	24.95	24.98	0.788	0.490
Total duration of pregnancies (average, in months)		1192	14.52	15.93	15.22	0.237	0.103
Breast feeding (%)		1147	55.4%	61.3%	58.0%	0.161	0.070
Total duration of breast feeding (average, in months)		1147	4.88	5.72	6.43	0.206	0.097
Died during follow-up (%)		484	17.4%	16.9%	13.2%	0.760	0.686

SNP: rs12443621 near TNRC9

Patient or disease characteristics		n=	Phenotype per genotype			p-value 3 groups[1]	p-value 2 groups[2]
			wt/wt	wt/mt	mt/mt		
Genotype (number of patients)			403	688	244		
Age at diagnosis (average)		1231	54.08	53.81	53.45	0.800	0.596
Age groups at diagnosis (%)	<40 years	1230	10.9%	11.0%	11.4%	0.625	0.932
	40-50 years		25.5%	26.3%	25.0%		
	50-60 years		33.5%	33.0%	39.5%		
	>60 years		30.1%	29.7%	24.1%		
BMI (average)		1186	25.78	25.11	25.01	0.043	0.015
Bilateral breast cancer present (%)		1242	6.9%	5.3%	8.1%	0.297	0.577
Stage of primary tumor (%)	T0 (in situ)	1216	6.8%	9.2%	13.4%	0.344	0.399
	T1		58.3%	56.0%	52.5%		
	T2		27.6%	27.3%	29.0%		
	T3 or T4		7.4%	7.5%	5.1%		
Lymph nodes involved (stage N1, N2 or N3) (%)		1103	39.5%	42.4%	41.3%	0.698	0.412
Clinical UICC stage (%)	1	1069	45.9%	43.1%	44.8%	0.740	0.769
	2		46.8%	48.7%	49.7%		
	3		7.3%	8.2%	5.5%		
Bloom-Richardson grade (%)	1	947	17.7%	18.8%	18.1%	0.970	0.808
	2		42.8%	40.7%	40.0%		
	3		39.6%	40.5%	41.9%		
Progesterone receptor positive (%)		673	61.8%	58.6%	47.7%	0.048	0.158
Estrogen receptor positive (%)		818	72.4%	72.2%	68.6%	0.689	0.748
Family history positive for breast cancer (%)		1229	43.1%	36.5%	43.6%	0.052	0.112
Family history positive for breast and/or ovarian cancer (%)		1229	45.5%	38.4%	44.5%	0.055	0.069
Number of relatives with breast cancer divided by total number of female relatives (average)		1229	0.064	0.065	0.078	0.270	0.457
Number of relatives with breast or ovarian cancer divided by total number of female relatives (average)		1229	0.071	0.070	0.081	0.471	0.808
Number of first degree relatives with breast cancer (average)		1229	0.23	0.24	0.32	0.106	0.326
Ever pregnant (%)		1157	80.6%	81.9%	84.9%	0.449	0.415
Age at first pregnancy (average)		941	25.48	24.82	25.15	0.147	0.092
Total duration of pregnancies (average, in months)		1192	14.64	15.21	15.10	0.813	0.525
Breast feeding (%)		1192	52.2%	59.2%	59.5%	0.074	0.023
Total duration of breast feeding (average, in months)		1192	4.78	5.63	4.93	0.370	0.273
Died during follow-up (%)		488	17.5%	17.6%	15.0%	0.836	0.858

SNP: rs8051542 near TNRC9

Patient or disease characteristics		n=	Phenotype per genotype			p-value 3 groups[1]	p-value 2 groups[2]
			wt/wt	wt/mt	mt/mt		
Genotype (number of patients)			331	601	264		
Age at diagnosis (average)		1094	53.37	53.55	55.27	0.093	0.340
Age groups at diagnosis (%)	<40 years	1094	10.4%	12.7%	7.9%	0.464	0.827
	40-50 years		25.2%	25.9%	24.4%		
	50-60 years		36.6%	32.5%	36.4%		
	>60 years		27.9%	28.9%	31.4%		
BMI (average)		1055	25.54	25.19	25.42	0.522	0.355
Bilateral breast cancer present (%)		1105	6.9%	6.1%	6.5%	0.892	0.674
Stage of primary tumor (%)	T0 (in situ)	1082	8.1%	8.8%	11.6%	0.446	0.557
	T1		57.6%	56.0%	53.9%		
	T2		28.3%	26.7%	29.5%		
	T3 or T4		6.1%	8.5%	5.0%		
Lymph nodes involved (stage N1, N2 or N3) (%)		983	43.7%	40.9%	40.1%	0.676	0.390
Clinical UICC stage (%)	1	951	42.7%	44.3%	45.7%	0.173	0.114
	2		52.3%	46.4%	48.1%		
	3		5.0%	9.4%	6.3%		
Bloom-Richardson grade (%)	1	846	15.5%	18.0%	19.8%	0.310	0.567
	2		44.0%	44.0%	35.8%		
	3		40.5%	37.9%	44.4%		
Progesterone receptor positive (%)		618	62.2%	58.1%	55.3%	0.459	0.260
Estrogen receptor positive (%)		736	70.0%	76.5%	66.9%	0.051	0.301
Family history positive for breast cancer (%)		1092	40.4%	41.5%	39.5%	0.863	0.887
Family history positive for breast and/or ovarian cancer (%)		1092	43.1%	43.5%	41.2%	0.826	0.922
Number of relatives with breast cancer divided by total number of female relatives (average)		1092	0.069	0.072	0.062	0.480	0.985
Number of relatives with breast or ovarian cancer divided by total number of female relatives (average)		1092	0.076	0.077	0.066	0.400	0.743
Number of first degree relatives with breast cancer (average)		1092	0.26	0.27	0.23	0.688	0.977
Ever pregnant (%)		1027	82.1%	83.4%	81.7%	0.832	0.785
Age at first pregnancy (average)		841	24.90	25.09	25.24	0.749	0.505
Total duration of pregnancies (average, in months)		1056	15.74	14.99	14.65	0.620	0.356
Breast feeding (%)		1056	55.4%	58.9%	57.3%	0.613	0.372
Total duration of breast feeding (average, in months)		1056	4.87	5.35	6.04	0.428	0.327
Died during follow-up (%)		464	12.6%	16.8%	18.0%	0.465	0.228

SNP: rs889312 near MAP3K1

Patient or disease characteristics		n=	Phenotype per genotype			p-value 3 groups[1]	p-value 2 groups[2]
			wt/wt	wt/mt	mt/mt		
Genotype (number of patients)			624	581	131		
Age at diagnosis (average)		1232	53.95	53.66	52.93	0.656	0.512
Age groups at diagnosis (%)	<40 years	1232	11.1%	11.2%	11.6%	0.861	0.994
	40-50 years		26.3%	25.0%	31.4%		
	50-60 years		33.9%	34.8%	33.1%		
	>60 years		28.7%	29.0%	24.0%		
BMI (average)		1186	25.23	25.46	24.87	0.387	0.640
Bilateral breast cancer present (%)		1243	6.9%	5.5%	6.6%	0.615	0.357
Stage of primary tumor (%)	T0 (in situ)	1217	9.0%	10.1%	9.2%	0.447	0.176
	T1		53.8%	56.3%	62.2%		
	T2		30.1%	26.5%	22.7%		
	T3 or T4		7.1%	7.1%	5.9%		
Lymph nodes involved (stage N1, N2 or N3) (%)		1102	45.0%	39.5%	35.8%	0.090	0.044
Clinical UICC stage (%)	1	1066	42.2%	44.0%	50.5%	0.641	0.591
	2		49.8%	48.8%	42.9%		
	3		8.0%	7.2%	6.7%		
Bloom-Richardson grade (%)	1	947	18.8%	16.0%	23.9%	0.256	0.353
	2		38.9%	44.5%	39.1%		
	3		42.3%	39.5%	37.0%		
Progesterone receptor positive (%)		672	56.2%	57.9%	64.1%	0.516	0.481
Estrogen receptor positive (%)		818	68.3%	75.1%	70.7%	0.119	0.056
Family history positive for breast cancer (%)		1230	38.9%	41.0%	42.5%	0.663	0.393
Family history positive for breast and/or ovarian cancer (%)		1230	40.8%	42.9%	45.0%	0.627	0.387
Number of relatives with breast cancer divided by total number of female relatives (average)		1230	0.068	0.066	0.071	0.874	0.832
Number of relatives with breast or ovarian cancer divided by total number of female relatives (average)		1230	0.075	0.070	0.080	0.659	0.713
Number of first degree relatives with breast cancer (average)		1230	0.26	0.24	0.25	0.909	0.676
Ever pregnant (%)		1156	82.0%	81.8%	85.7%	0.602	0.841
Age at first pregnancy (average)		943	24.78	25.40	24.85	0.115	0.075
Total duration of pregnancies (average, in months)		1192	15.27	14.45	16.23	0.353	0.522
Breast feeding (%)		1192	55.6%	57.2%	60.3%	0.627	0.455
Total duration of breast feeding (average, in months)		1192	5.20	5.29	5.14	0.984	0.911
Died during follow-up (%)		492	17.1%	18.1%	13.0%	0.670	0.991

SNP: rs13281615 on 8q24

Patient or disease characteristics		n=	Phenotype per genotype			p-value 3 groups[1]	p-value 2 groups[2]
			wt/wt	wt/mt	mt/mt		
Genotype (number of patients)			440	692	213		
Age at diagnosis (average)		1241	54.01	53.56	54.00	0.784	0.612
Age groups at diagnosis (%)	<40 years	1240	10.9%	11.3%	10.9%	0.926	0.866
	40-50 years		24.6%	27.5%	23.8%		
	50-60 years		35.0%	33.5%	35.8%		
	>60 years		29.4%	27.7%	29.5%		
BMI (average)		1194	25.27	25.15	25.77	0.248	0.953
Bilateral breast cancer present (%)		1252	6.6%	6.5%	4.6%	0.578	0.717
Stage of primary tumor (%)	T0 (in situ)	1227	9.5%	9.7%	9.3%	0.272	0.498
	T1		54.9%	54.9%	61.9%		
	T2		27.4%	29.4%	22.2%		
	T3 or T4		8.2%	6.0%	6.7%		
Lymph nodes involved (stage N1, N2 or N3) (%)		1111	42.8%	43.6%	32.4%	0.030	0.570
Clinical UICC stage (%)	1	1076	42.3%	42.5%	53.3%	0.041	0.188
	2		48.3%	51.2%	40.1%		
	3		9.4%	6.3%	6.6%		
Bloom-Richardson grade (%)	1	955	16.4%	18.2%	22.1%	0.674	0.598
	2		42.1%	41.6%	38.3%		
	3		41.5%	40.2%	39.6%		
Progesterone receptor positive (%)		674	54.1%	59.1%	63.2%	0.271	0.145
Estrogen receptor positive (%)		822	68.3%	72.5%	78.3%	0.130	0.106
Family history positive for breast cancer (%)		1239	39.3%	40.0%	41.2%	0.903	0.748
Family history positive for breast and/or ovarian cancer (%)		1239	41.8%	42.0%	41.8%	0.996	0.952
Number of relatives with breast cancer divided by total number of female relatives (average)		1239	0.064	0.067	0.073	0.632	0.512
Number of relatives with breast or ovarian cancer divided by total number of female relatives (average)		1239	0.071	0.074	0.075	0.872	0.628
Number of first degree relatives with breast cancer (average)		1239	0.23	0.26	0.25	0.605	0.348
Ever pregnant (%)		1165	81.3%	81.9%	84.4%	0.644	0.613
Age at first pregnancy (average)		948	25.33	24.74	25.47	0.091	0.182
Total duration of pregnancies (average, in months)		1201	14.34	15.62	14.34	0.258	0.234
Breast feeding (%)		1201	57.9%	56.5%	55.9%	0.864	0.600
Total duration of breast feeding (average, in months)		1201	5.41	5.22	4.92	0.853	0.666
Died during follow-up (%)		490	19.3%	17.3%	10.8%	0.280	0.340

SNP: rs3817198 in LSP1

Patient or disease characteristics		n=	Phenotype per genotype			p-value 3 groups[1]	p-value 2 groups[2]
			wt/wt	wt/mt	mt/mt		
Genotype (number of patients)			633	574	126		
Age at diagnosis (average)		1232	53.62	54.08	53.13	0.640	0.652
Age groups at diagnosis (%)	<40 years	1231	11.6%	10.4%	12.1%	0.918	0.846
	40-50 years		26.2%	24.7%	28.4%		
	50-60 years		35.0%	35.1%	31.9%		
	>60 years		27.2%	29.8%	27.6%		
BMI (average)		1187	25.27	25.33	24.98	0.750	0.970
Bilateral breast cancer present (%)		1243	5.9%	5.8%	10.2%	0.194	0.615
Stage of primary tumor (%)	T0 (in situ)	1219	10.0%	9.2%	8.5%	0.534	0.214
	T1		56.4%	55.7%	55.1%		
	T2		27.4%	27.1%	29.7%		
	T3 or T4		6.2%	8.1%	6.8%		
Lymph nodes involved (stage N1, N2 or N3) (%)		1102	38.7%	44.0%	44.4%	0.190	0.078
Clinical UICC stage (%)	1	1068	44.4%	45.2%	39.6%	0.557	0.489
	2		49.0%	46.1%	52.8%		
	3		6.6%	8.7%	7.5%		
Bloom-Richardson grade (%)	1	948	19.1%	17.4%	16.5%	0.917	0.724
	2		41.0%	40.3%	43.5%		
	3		39.9%	42.3%	40.0%		
Progesterone receptor positive (%)		672	60.8%	55.7%	54.5%	0.375	0.164
Estrogen receptor positive (%)		817	71.9%	72.0%	68.4%	0.812	0.870
Family history positive for breast cancer (%)		1230	38.0%	41.5%	43.1%	0.382	0.177
Family history positive for breast and/or ovarian cancer (%)		1230	39.7%	43.8%	44.8%	0.312	0.130
Number of relatives with breast cancer divided by total number of female relatives (average)		1230	0.063	0.072	0.067	0.416	0.215
Number of relatives with breast or ovarian cancer divided by total number of female relatives (average)		1230	0.068	0.078	0.075	0.351	0.153
Number of first degree relatives with breast cancer (average)		1230	0.23	0.28	0.22	0.298	0.276
Ever pregnant (%)		1156	81.7%	80.3%	89.3%	0.090	0.901
Age at first pregnancy (average)		938	24.80	25.22	25.62	0.176	0.090
Total duration of pregnancies (average, in months)		1192	15.32	14.26	16.63	0.166	0.419
Breast feeding (%)		1192	58.8%	54.9%	56.5%	0.428	0.207
Total duration of breast feeding (average, in months)		1192	5.43	5.07	5.01	0.809	0.517
Died during follow-up (%)		485	15.0%	19.4%	18.2%	0.466	0.222

In this supplemental table, the associations of SNP genotypes with disease and patients characteristics are explored in the ORIGO cohort. The following information is given for each patient characteristic: the total number of patients for whom this characteristic was known, the distribution or average of this characteristic or phenotype per genotype, and the p-values comparing the three genotype groups separately or in a co-dominant model.
Abbreviations: SNP, single nucleotide polymorphism; wt/wt, homozygotes for the wildtype allele; wt/mt, heterozygotes; mt/mt, homozygotes for the minor allele.
[1] p-value comparing the three genotype groups separately
[2] p-value in a co-dominant model, comparing the group of homozygotes for the wildtype allele with the combined group of heterozygotes and homozygotes for the minor allele
